# Angiosarcoma on Untreated Facial Capillary Malformations

**Published:** 2015-06-22

**Authors:** Akimitsu Nishibayashi, Yuki Hata, Yumiko Hori, Koichi Tomita, Ken Matsuda, Kenji Yano, Ko Hosokawa

**Affiliations:** ^a^Department of Plastic and Reconstructive Surgery, Osaka University Graduate School of Medicine, Yamadaoka, Suita-city, Osaka, Japan; ^b^Department of Pathology, Osaka University Graduate School of Medicine, Yamadaoka, Suita-city, Osaka, Japan

**Keywords:** anigosarcoma, capillary malformation, bleeding, ulcer, free flap

## DESCRIPTION

**Figure 1:**
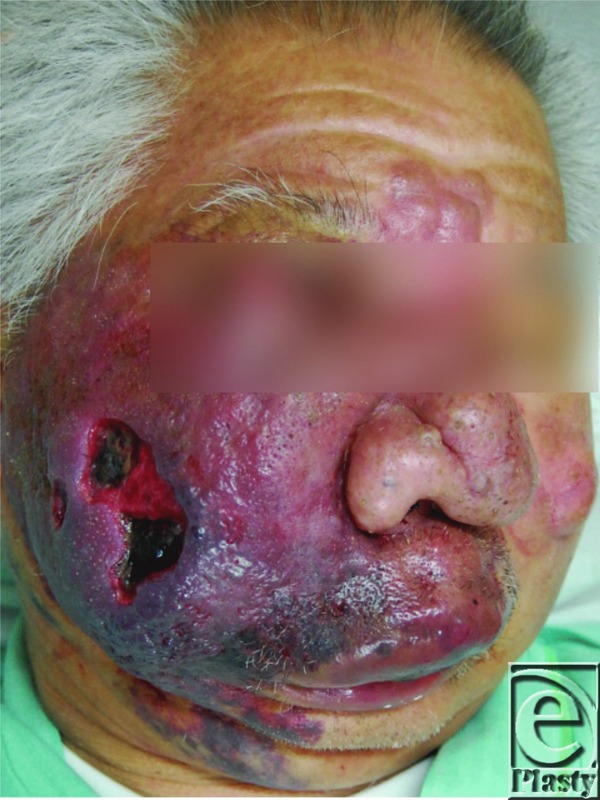
Bleeding from the ulcer on the center of the port-wine stain on the cheek temporarily stopped.

**Figure 2:**
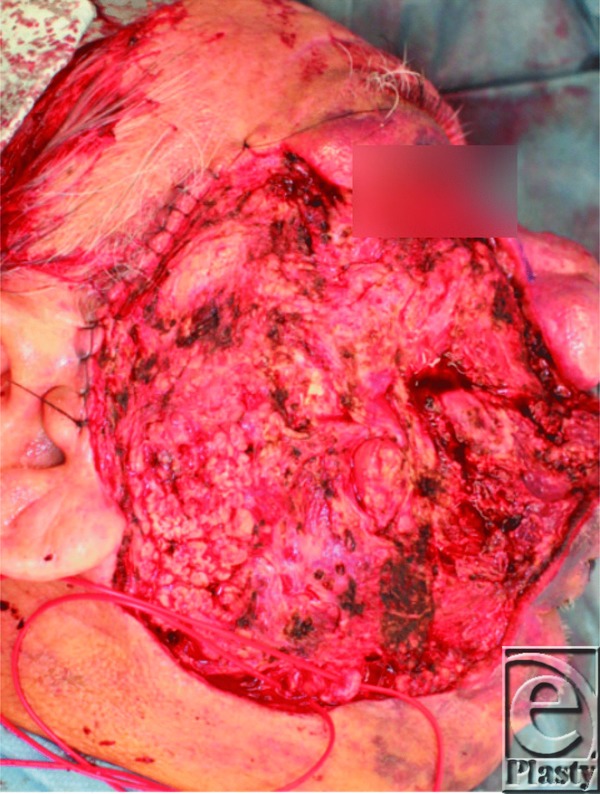
Resection of the port-wine stain. The facial artery and vein were exposed for microvascular reconstruction.

**Figure 3:**
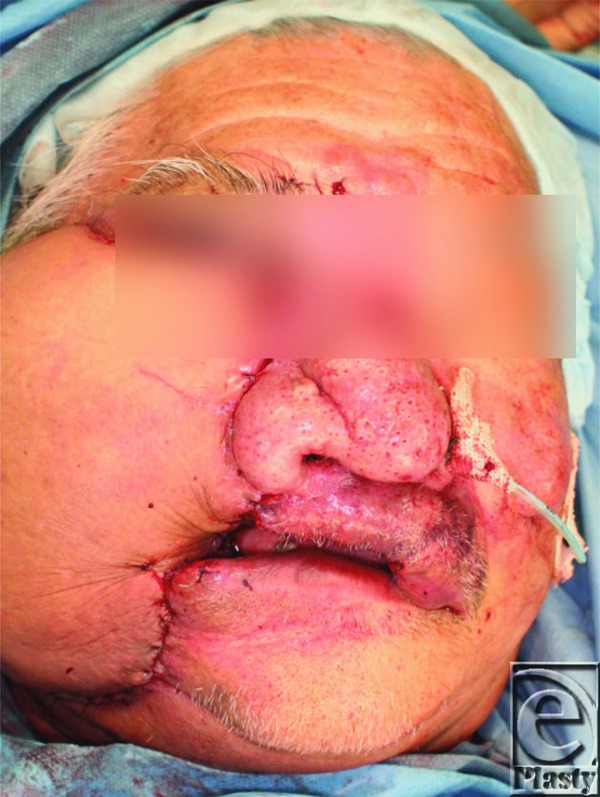
After reconstruction using an anterolateral thigh flap. The flap covered the entire raw surface but was swelled and bulky.

A 73-year-old man with a right facial capillary malformation with ulcerations and uncontrollable oozing was referred to our institution. Bleeding was temporarily controlled but could not be stopped (Fig 1). Anemia worsened daily, leading us to resect the port-wine stain on the right cheek (Fig 2). The subcutaneous defect was covered with a free anterolateral thigh flap (Fig 3). Bleeding stopped 1 week postoperatively. However, a new ulcer, identified as an angiosarcoma, appeared on the residual port-wine stain on the lip and bleeding resumed. The patient refused additional treatment and died of blood loss 38 days postoperatively.

## QUESTIONS

1. **What is the risk and epidemiology of angiosarcomas?**
2. **What margins of resection are required to excise an angiosarcoma?**
3. **What treatment options other than surgery are available?**
4. **What are the options for reconstruction when most of the facial soft tissue is removed from one side?**

## DISCUSSION

Angiosarcomas are rare tumors that typically occur in the head and neck regions of elderly male patients and are classified into normal, nodular, and ulcer types. They account for roughly 15% of all head and neck sarcomas and 1% of all soft-tissue sarcomas.^1^^,^^2^ Generally, age of incidence and localization to the face or scalp have led to the proposal that sun exposure contributes to the cause, but this has not been clearly demonstrated. Five percent to 20% of patients have a history of radiotherapy to the face or the scalp.^3^ Only a few reports exist in the literature regarding angiosarcomas occurring on capillary malformations, with few details provided.^4^

Complete resection of these lesions is very difficult, as is the case for other malignant facial tumors. Complete surgical resection with wide margins is preferred for localized disease, although the safety margin for excising angiosarcomas is not clear. One study reported that a margin greater than 3 cm wide is necessary for high-grade sarcomas.^5^ But this is not feasible on the face or scalp. Complete resection was particularly difficult in our case patient because the angiosarcoma occurred on a capillary malformation, making it challenging to identify the border between normal tissue and the malignant tumor. Angiosarcomas are often locally advanced at presentation, and, in some cases, the lesion is too broad to resect completely.

Treatments of angiosarcomas include surgical resection with a wide margin and radiotherapy. The efficacy of chemotherapy is unclear.^6^ However, radiotherapy and chemotherapy are options for patients with unresectable lesions or for those who refuse surgery. According to one report, angiosarcomas are particularly responsive to taxane.^7^

There are several ways to reconstruct a huge facial defect after resection of facial soft tissue (eg, full-thickness skin graft, split-thickness skin graft, free flap transfer). Bleeding underneath the skin graft prevents adaptation. In our case, the defect was very deep and prone to bleeding, leading to the use of an anterolateral thigh flap. Unfortunately, the color did not match due to differences in the color of the thigh and face. Kim et al^8^ reported that a split-thickness skin graft is an option for wide wounds after the resection of facial capillary malformations. If bleeding can be completely controlled, a split-thickness skin graft may be a good option.

Facial capillary malformations become darker with age and can occasionally progress and lead to severe bleeding. This may complicate the diagnosis of angiosarcomas on capillary malformations. The diagnosis of angiosarcoma was delayed in our patient, in part, because circulatory failure of hypertrophic soft tissue on the port-wine stain was considered to be the cause of the bleeding ulceration, rather than a malignant neoplasm. Given that little is known about angiosarcomas, their treatment remains challenging.
